# *Propionibacterium freudenreichii* thrives in microaerobic conditions by complete oxidation of lactate to CO_2_

**DOI:** 10.1111/1462-2920.15532

**Published:** 2021-05-06

**Authors:** Alexander Dank, Oscar van Mastrigt, Sjef Boeren, Søren K. Lillevang, Tjakko Abee, Eddy J. Smid

**Affiliations:** ^1^ Laboratory of Food Microbiology Wageningen University & Research, P.O. Box 17 Wageningen 6700AA The Netherlands; ^2^ Laboratory of Biochemistry Wageningen University & Research Wageningen The Netherlands; ^3^ Arla Innovation Centre, Arla Foods, Agro Food Park 19 Aarhus N 8200 Denmark

## Abstract

In this study we show increased biomass formation for four species of food‐grade propionic acid bacteria (*Acidipropionibacterium acidipropionici*, *Acidipropionibacterium jensenii*, *Acidipropionibacterium thoenii* and *Propionibacterium freudenreichii*) when exposed to oxygen, implicating functional respiratory systems. Using an optimal microaerobic condition, *P*. *freudenreichii* DSM 20271 consumed lactate to produce propionate and acetate initially. When lactate was depleted propionate was oxidized to acetate. We propose to name the switch from propionate production to consumption in microaerobic conditions the ‘propionate switch’. When propionate was depleted the ‘acetate switch’ occurred, resulting in complete consumption of acetate. Both growth rate on lactate (0.100 versus 0.078 h^−1^) and biomass yield (20.5 versus 8.6 g* mol^−1^ lactate) increased compared to anaerobic conditions. Proteome analysis revealed that the abundance of proteins involved in the aerobic and anaerobic electron transport chains and major metabolic pathways did not significantly differ between anaerobic and microaerobic conditions. This implicates that *P*. *freudenreichii* is prepared for utilizing O_2_ when it comes available in anaerobic conditions. The ecological niche of propionic acid bacteria can conceivably be extended to environments with oxygen gradients from oxic to anoxic, so‐called microoxic environments, as found in the rumen, gut and soils, where they can thrive by utilizing low concentrations of oxygen.

## Introduction

Propionic acid bacteria (PAB) are Gram‐positive, non‐spore‐forming bacteria belonging to the group of actinobacteria with a high GC content (53%–68%) (Poonam *et al*., 2012). PAB belong to the family of *Propionibacteriaceae* and were historically classified based on their habitat as classical (dairy) or cutaneous PAB. However, later Scholz and Kilian (2016) proposed to reclassify cutaneous and dairy PAB based on whole‐genome sequence analysis of 162 strains of the family *Propionibacteriaceae* by addition of three novel genera: *Acidipropionibacterium*, *Cutibacterium* and *Pseudopropionibacterium*. Major species of dairy PAB that are isolated mainly from milk, cheese, dairy products and rumen are *Propionibacterium freudenreichii*, *Acidipropionibacterium acidipropionici*, *Acidipropionibacterium jensenii* and *Acidipropionibacterium thoenii* (Poonam *et al*., 2012).

Notably, PAB can be found in a large variety of environments, ranging from the deepest cave of the world (Kieraite‐Aleksandrova *et al*., 2015), soil (Hayashi and Furusaka, 1979), silage (Merry and Davies, 1999), human skin (Perry and Lambert, 2006) and other tissue (Perry and Lambert, 2011), cheese (Britz and Riedel, 1994) and the rumen of animals (Bryant, 1959). This variety of environments points to versatility in metabolic traits of which the repertoire can be extended by the capacity to use a range of terminal electron acceptors. Indeed, PAB have been reported to use several alternative electron acceptors, like nitrate (Kaspar, 1982; Allison and Macfarlane, 1989) and humic acid (Benz *et al*., 1998) next to oxygen (van Gent‐Ruijters *et al*., 1976). The complete TCA cycle and genes corresponding to respiration pathways and electron transport chains were found in *Acidipropionibacterium acidipropionici* (Parizzi *et al*., 2012) and *Propionibacterium freudenreichii* (Falentin *et al*., 2010). Although their uses are mainly in anaerobic processes, the presence of all genes required for respiration and fully functional electron transport chains (Falentin *et al*., 2010; Parizzi *et al*., 2012) shows the potential for applications in (micro)aerobic biotechnological processes. However, studies addressing the effect of oxygen on PAB present contradicting results. An increased growth rate and biomass production are reported for *P*. *freudenreichii* (Quesada‐Chanto *et al*., 1998; Cardoso *et al*., 2004), showing the potential benefit of using oxygen as a terminal electron acceptor. However, also the inability of *P*. *freudenreichii* to grow aerobically on agar plates, lower growth rates (de Vries *et al*., 1972; Ye *et al*., 1996) and decrease of cytochrome synthesis and consequent loss of electron transport chain integrity are reported under aerobic conditions (de Vries *et al*., 1972), which shows the sensitivity of *P*. *freudenreichii* to high levels of oxygen. Oxygen toxicity can be combatted by PAB by expressing superoxide dismutase, catalase and cytochrome *c* oxidase, although the distribution of these enzymes seems to be species‐specific (Cove *et al*., 1987). The relationship of various species of PAB with oxygen is thus complex and requires further study.

The aims of this study are twofold. First, we determined biomass formation and metabolite production of food‐grade PAB strains as a function of various exposure to oxygen. The food‐grade PAB strains are represented by the species *A*. *acidipropionici*, *A*. *jensenii*, *A*. *thoenii*, *P*. *freudenreichii* subsp. *freudenreichii* and *P*. *freudenreichii* subsp. *shermanii*. Second, the commonly used dairy isolate *P*. *freudenreichii* DSM 20271 was selected for an in‐depth study of its response to oxygen. An optimum oxygen flux was determined in chemostat cultivations and using this oxygen concentration the biomass production, primary metabolite production and proteome were monitored during long‐term cultivation. Our study provides implications for PAB ecology as well as industrial applications including increased biomass production and yield enhancements for efficient production of PAB adjunct cultures and/or for various biotechnological purposes, like vitamin and aroma production.

## Results and discussion

### Screening of response to oxygen for 16 propionic acid bacteria

**Fig. 1:**
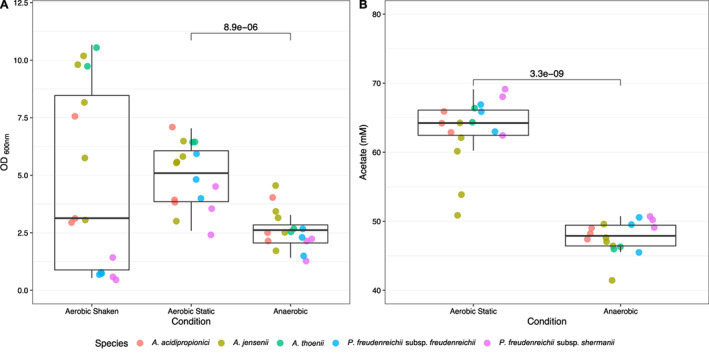
Biomass production (A) and acetate production (B) for four species of propionic acid bacteria of cells growing in aerobic shaking (120 rpm), aerobic static and anaerobic conditions. Each data point represents the average of biological duplicates for each individual strain (*n* = 16 strains). [Color figure can be viewed at wileyonlinelibrary.com]

In this study we screened the response to various levels of oxygen for 16 PAB strains (Supplementary File 1). Biomass formation (optical density at OD_600nm_) was determined for each strain after 5 days of incubation in yeast extract lactate (YEL) medium in three conditions: (i) aerobic shaking (120 rpm), (ii) aerobic static and (iii) anaerobic. Anaerobic cultivation resulted in a mean OD_600nm_ value of 2.6 ± 0.2, aerobic static in a mean OD_600nm_ of 5.0 ± 0.3 and aerobic shaken in a mean OD_600nm_ of 4.8 ± 1.0 (Fig. 1A). A significant difference between the OD_600nm_ values was found between anaerobic and aerobic static (*P* < 1E^−5^, Pairwise Wilcoxon test).

The availability of oxygen enhanced biomass formation in static conditions for all strains, although the strain‐to‐strain variation was enlarged. In aerobic shaken conditions the increased oxygen levels resulted in lower biomass production of the selected *P*. *freudenreichii* subsp. *freudenreichii* and *P*. *freudenreichii* subsp. *shermanii* strains compared to anaerobic conditions. Several *A*. *acidipropionici*, *A*. *thoenii* and *A*. *jensenii* strains were able to produce similar or higher biomass compared to anaerobic conditions and thus are more tolerant to higher oxygen levels.

Metabolite formation was determined for aerobic static and anaerobic conditions. The presence of oxygen resulted in a significantly higher amount of acetate being produced by strains incubated in aerobic static conditions (*P* < 1e^−8^_,_ Wilcoxon rank‐sum test) compared to anaerobic conditions (Fig. 1B). Higher acetate production in aerobic conditions provides evidence that active electron transport using oxygen as terminal electron acceptor is widespread in PAB. The higher biomass formation shows that PAB can energetically benefit from respiratory electron transport, in line with the genomic information available for *P*. *freudenreichii* (Falentin *et al*., 2010) and *A*. *acidipropionici* (Parizzi *et al*., 2012). However, our results also clearly demonstrate the toxicity of high oxygen levels for certain PAB in aerobic shaking conditions.

Inter‐ and intra‐species differences in the aerotolerance of PAB may be explained by variability in oxygen‐defence systems (Cove *et al*., 1987), hence each individual strain must have an optimal level of oxygen at which it can combat oxygen toxicity and have maximal energetic benefit. We focussed further on studying the effect of oxygen on the commonly used cheese adjunct culture *P*. *freudenreichii* DSM 20271, which obtained an OD_600nm_ of 4.0 in aerobic static conditions and an OD_600nm_ of 0.9 in aerobic shaken conditions, clearly showing both the growth stimulation effect of O_2_ and its toxicity at higher exposure. In order to find the optimal oxygen concentration for *P*. *freudenreichii* DSM 20271, we performed chemostat cultivations at pH 7.0 at a constant dilution rate of 0.1 h^−1^, while varying the amount of oxygen supplied to the system. The highest concentrations of biomass were formed using oxygen supplies of 4.2 and 8.4 mL O_2_*L medium^−1^*min^−1^. At oxygen supplies above 8.4 mL O_2_*L medium^−1^*min^−1^ the biomass formation showed a sharp decline (Supplementary Fig. 1). In further experiments, an oxygen supply of 6.3 mL O_2_*L medium^−1^*min^−1^ was used to study the growth, metabolite production and proteome of *P*. *freudenreichii* DSM 20271.

### Microaerobic conditions increase biomass production in prolonged batch cultivations by complete oxidation of lactate

#### Metabolite production and biomass formation

**Fig. 2:**
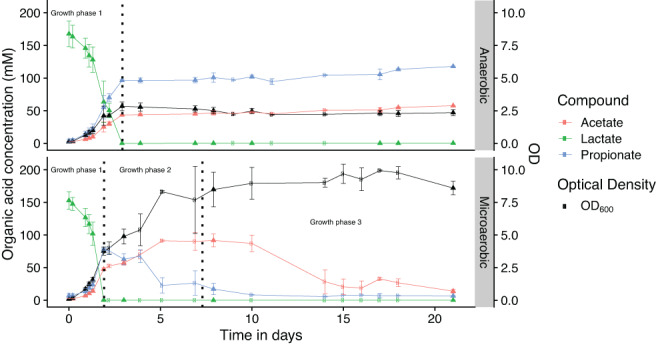
Metabolite production and biomass production (black line) of *P*. *freudenreichii* DSM 20271 during cultivation under anaerobic and microaerobic (6.3 mL O_2_*L medium^−1^*min^−1^) conditions over a course of 21 days. Three clear phases can be distinguished: Growth phase 1 – Lactate consumption and propionate and acetate formation. Growth phase 2 – Propionate conversion to acetate and CO_2_. Phase 3 – Acetate oxidation to CO_2_. Error bars present the standard error of biological replicates. Biological replicates are displayed by either triangles (*n* = 3) or circles (*n* = 2). [Color figure can be viewed at wileyonlinelibrary.com]

*Propionibacterium freudenreichii* DSM 20271 was cultured in a prolonged batch cultivation in pH and temperature‐controlled bioreactors for a period of 21 days. Biomass production, primary metabolite production (Fig. 2) and proteomes were monitored. In anaerobic conditions lactate was consumed within 72 h and a stable propionate:acetate ratio of 2.1:1 was found throughout the duration of the cultivation after lactate depletion. Without additional external electron acceptors *P*. *freudenreichii* is thus unable to further degrade these organic acids.

In microaerobic conditions lactate was completely oxidized in three distinguishable phases, (i) Lactate was consumed and propionate and acetate were produced, (ii) propionate was oxidized to acetate and (iii) acetate was completely oxidized to CO_2_ (Fig. 2). Oxygen was actively consumed throughout the cultivation, as dissolved oxygen levels dropped below the detection limit 1 day after inoculation and remained undetectable until all propionate was consumed. Oxygen levels also remained undetectable during acetate consumption, but increased again towards the end of the acetate consumption phase, signifying a reduction in respiration rate (Supplementary Fig. 2). Metabolite production and biomass formation in each phase is discussed below.

##### Lactate consumption phase

In microaerobic conditions, lactate was consumed within 46 h and propionate and acetate were formed in a ratio of 1.56:1. A significantly higher maximum growth rate (0.100 ± 0.002 h^−1^ versus 0.078 ± 0.004 h^−1^, one‐tailed *t*‐test, *P* < 0.01) and higher biomass (OD_600nm_ of 3.7 ± 0.5 versus 2.9 ± 0.6, one‐tailed *t*‐test, *P* < 0.05) were achieved in the lactate consumption phase (phase 1) for cells grown microaerobically compared to anaerobically grown cells. Anaerobic fermentation of lactate resulted in an average yield of 8.6 ± 1.2 g cell dry weight* mol^−1^ lactate (Table 1), in line with the yield of 8.1 g cell dry weight* mol^−1^lactate reported by de Vries *et al*. (1973) for anaerobic growth on a synthetic medium but low compared to the yield of 10.2 on complex lactate medium. Differences in yeast extract content may account for the different yield reported by de Vries *et al*. (1973). Microaerobic conditions yielded 11.6 ± 0.7 g cell dry weight* mol^−1^ lactate, a 1.3‐fold increase compared to anaerobic conditions. The production of propionate in microaerobic conditions implies that the flux through the electron transport chain is limited or stoichiometrically constrained by the availability of oxygen, resulting in propionate production as the main electron sink.

**Table 1:** Calculated cell dry weight, summation of cell yield after each phase and maximum growth rate of *P*. *freudenreichii* grown in anaerobic or microaerobic conditions.

Condition	Cell dry weight (g* kg^−1^)	Yield (g CDW* mol^‐1^)	Maximum growth rate (h^−1^)	
Microaerobic		Lactate consumption phase		
	1.8 ± 0.4	11.6 ± 0.7	0.100 ± 0.001	
		Propionate consumption phase		
	3.1 ± 0.10	20.5 ± 2.3	0.008 ± 0.003	
		Acetate consumption phase		
	3.1 ± 0.03	20.3 ± 1.8		
Anaerobic	Lactate consumption phase			
	1.4 ± 0.02	8.6 ± 1.2	0.078 ± 0.004

##### Propionate consumption phase

As cells deplete their lactate pool, the secreted organic acids become a potential source of energy in the presence of external terminal electron acceptors. When lactate was depleted, a switch from production to consumption of propionate was observed in microaerobic conditions (phase 2). Propionate was depleted after 143 h and an increase of biomass from OD_600nm_ 3.7 ± 0.5 to 9.2 ± 1.1 was observed. Growth rates in the propionate consumption phase decreased to 0.008 ± 0.003 h^−1^. Microaerobic conditions yielded an average estimated biomass of 20.5 ± 2.3 g cell dry weight*mol^−1^ lactate after exhaustion of propionate, a 2.4‐fold increase compared to the yield observed on lactate in anaerobic conditions.

A switch from propionate production to consumption was also observed by Ye *et al*. (1996) after changing from anaerobic to aerobic conditions in *P*. *freudenreichii*. *Escherichia coli* switches from production towards dissimilation of secreted acetate (‘the acetate switch’) when its preferred substrate becomes limiting and electron transport is possible (Wolfe, 2005). In analogy to this ‘acetate switch’, we propose to name the change from production to consumption of propionate when lactate is depleted in presence of electron acceptors in *P*. *freudenreichii* the ‘propionate switch’.

##### Acetate consumption phase

Interestingly, the addition of diluted hydrochloric acid to control the pH in the bioreactor continued from this point onwards, indicating consumption of organic acids. Indeed acetate consumption was observed after depletion of propionate (phase 3). Acetate was consumed and no other organic acids were detected, indicating complete oxidation to CO_2_. Beck and Schink (1995) showed *P*. *freudenreichii* DSM 20271 is able to completely oxidize acetate through a modified citric acid cycle using hexacyanoferrate as electron acceptor, supporting our findings. The oxidation of acetate was not accompanied by a further increase in OD_600nm_, in line with results of Beck and Schink (1995) who found linear growth kinetics and a maximum biomass increase of two. The linear growth kinetics observed on acetate could explain the preferred order of the ‘propionate switch’ with the production of acetate instead of complete combustion of propionate immediately. A schematic overview of the consumption phases based on proteome analysis and literature of *P*. *freudenreichii* metabolism (discussed below) is shown in Fig. 5.

In order to metabolize lactate, propionate and acetate as carbon sources, these compounds have to be transported into the cell. The undissociated form of these acids are membrane permeable (Salmond *et al*., 1984; Saparov *et al*., 2006) by passive diffusion. In our cultivations (pH 7.0) the main form of the acids is dissociated and these require active transport systems. The presence of a transporter of propionate and/or acetate would greatly increase the ability to grow on these substrates in environments with neutral pH. l‐lactate and d‐lactate can be transported into the cell by lactate permease (LldP) (Núñez *et al*., 2002), which has also been identified in the genome of *P*. *freudenreichii*. For propionate and acetate we did not find any annotated transporters in the genome of *P*. *freudenreichii*. Monocarboxylate uptake systems for propionate and acetate have been described for several other bacteria (Fernández‐Briera and Garrido‐Pertierra, 1988; Ebbighausen *et al*., 1991; Hosie *et al*., 2002; Gimenez *et al*., 2003; Reed *et al*., 2006) and yeast (Casal *et al*., 1996). Blasting JEN1, a high‐affinity symporter of lactate, pyruvate and acetate in *Saccharomyces cerevisiae*, resulted in a protein hit with 46% homology in *P*. *freudenreichii* annotated as Ydjk, a sugar (and other) transporter of unknown function which was not detected in our proteome analysis. In order to elucidate whether or not active transporters for propionate and/or acetate are present in *P*. *freudenreichii*, studies on the uptake rates of these monocarboxylates at different pH values need to be performed. This also further elucidates the effect of pH on the growth performance of *P*. *freudenreichii* on these monocarboxylates in the presence of external electron acceptors.

#### Bioenergetics

A functional respiratory chain greatly affects ATP production per substrate, as shown by the increased biomass yield in microaerobic conditions. Anaerobic growth on lactate using the Wood–Werkman cycle yields per three lactate, one ATP by substrate‐level phosphorylation and 2× 2/3 ATP by fumarate reduction (Seeliger *et al*., 2002). This totals to a maximum ATP yield of 0.78 mol ATP per mol lactate.

The modified citric acid cycle suggested by Beck and Schink (1995) generates three NAD(P)H and one menaquinol per cycle from acetyl‐CoA. Assuming lactate is converted to pyruvate by l‐lactate:menaquinone oxidoreductase (1.1.5.12), generating menaquinol, and pyruvate entering the TCA through acetyl‐CoA using pyruvate dehydrogenase (1.2.4.1/2.3.1.12/1.8.1.4), generating one NADH, a total yield of four NAD(P)H and two menaquinol per lactate is calculated.

The number of protons translocated across the membrane per electron transferred over an electron transport chain (H^+^/e^−^) consisting of a type‐I NADH dehydrogenase, a menaquinone pool and cytochrome bd oxidase is two (2H^+^/e^−^) per NADH oxidized, one by type‐I NADH dehydrogenase (Bongaerts *et al*., 1995) and one by the bd‐type cytochrome (Bott and Niebisch, 2003). Oxidation of menaquinol via bd‐type cytochrome results in one proton translocated per electron (1 H^+^/e^−^). Since two electrons are transferred in NADH oxidation and two electrons are transferred in menaquinol oxidation a total of 20 protons can be translocated per lactate. Succinate:menaquinone reductase requires a proton gradient to reduce menaquinone, which lowers the total potential ATP production by protons with two translocated protons that cannot be used by ATPase (Schirawski and Unden, 1998). A total of 18 protons per lactate oxidized are calculated. Assuming ATPase requiring three protons per ATP (Bott and Niebisch, 2003) a theoretical ATP yield of six ATP per mol lactate oxidized is calculated. Complete oxidation of lactate would thus maximally increase the ATP yield by a factor of 7.7 compared to the ATP yield by fermentation.

Our experimental results showed an increase in biomass of 2.4‐fold of cells in microaerobic conditions compared to anaerobic conditions, well within the limits of the maximum theoretical energetic advantage and similar to the biomass increase of 2.7 found by Pritchard *et al*. (1977) in chemostat conditions. The theoretical increase in biomass based on ATP generation difference is much higher (7.7 times). Partly, this may be explained by the expression of pyruvate oxidase (Marcellin *et al*., 2020). Our proteomics data showed pyruvate oxidase is expressed in anaerobic and microaerobic conditions and low oxygen contents did not significantly change expression. If pyruvate oxidase is used as an oxygen‐consuming defence mechanism in microaerobic conditions instead of pyruvate dehydrogenase or pyruvate ferredoxin oxidoreductase in anaerobic conditions, less reducing equivalents (one less NADH) are produced and the theoretical ATP yield is lowered by 1.33 ATP. However, the theoretical yield when assuming pyruvate oxidase activity is still much higher. Beck and Schink (1995) report sub‐exponential growth kinetics and as a consequence low biomass yields when acetate is oxidized by *P*. *freudenreichii*. We hypothesize that because of these sub‐exponential growth kinetics, generated energy from acetate oxidation is mainly directed towards maintenance processes.

#### Proteome analysis of long‐term batch cultivation

**Fig. 3:**
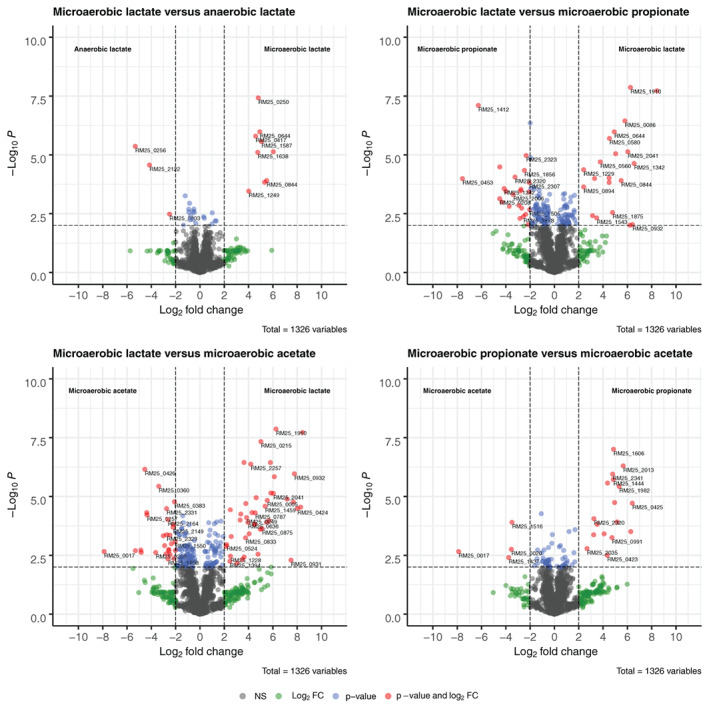
Volcano plots of proteomes measured during anaerobic and microaerobic growth conditions. A comparison is made between two phases: Reference versus treatment, positive log_2_ fold change values indicate higher abundance in the reference sample, negative log_2_ fold change values indicate higher abundance in the compared sample. Each point represents a unique protein. Colours represent significant differences between reference and treatment. Grey: Not significant (NS); green: >fourfold difference; blue: *P* < 0.01; red: >fourfold difference and *P* < 0.01 A list of significant proteins for each comparison is available in Supplementary File 2. [Color figure can be viewed at wileyonlinelibrary.com]

A proteomic analysis of cells in anaerobic and for each consumption phase in microaerobic conditions was performed. In total 1326 unique proteins were identified amongst all samples after strict filtering. For all identified proteins Volcano plots were prepared comparing the consumption phases and identifying significantly different abundant proteins (fourfold increase and *P* < 0.01 (Fig. 3)). A list with all significantly differentially expressed proteins for all conditions can be found in Supplementary File 2.

**Fig. 4:**
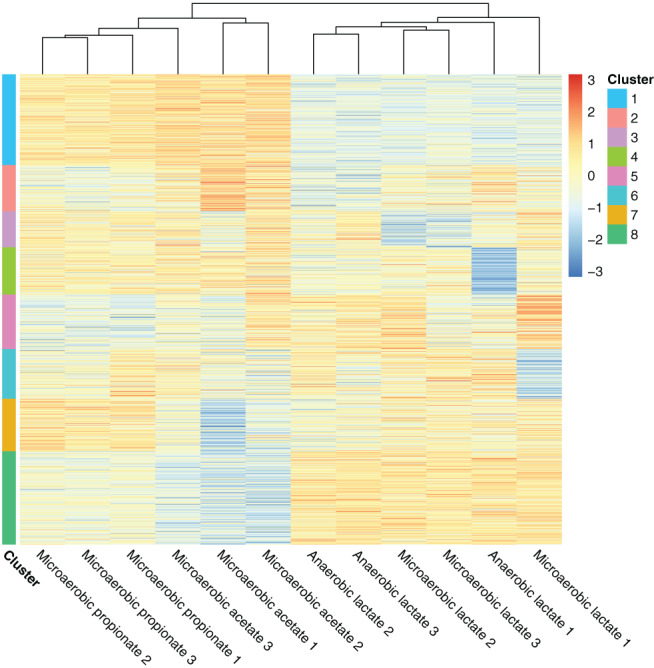
Hierarchical clustering of proteomes from cells growing in anaerobic or microaerobic conditions on lactate (anaerobic and microaerobic) and propionate and acetate (microaerobic). K‐means clustering with *k* = 8 was applied to cluster detected proteins into protein clusters with similar abundance patterns across each sample. Protein clusters can be found in Supplementary File 3. [Color figure can be viewed at wileyonlinelibrary.com]

To determine proteome similarity amongst samples and reveal protein expression patterns across samples we clustered the samples using hierarchical clustering and K‐means partitioning (MacQueen, 1967). Hierarchical clustering was used to cluster samples based on proteome similarity. K‐means partitioning was applied with *k* = 8 for identification of clusters of proteins with similar expression patterns across the samples (Karimpour‐Fard *et al*., 2015) (Fig. 4, Supplementary File 3). Cluster 2, 3, 4 and 6 did not show specific patterns amongst hierarchically grouped samples and thus most likely consists of proteins that are continuously expressed and which expression patterns were based on random variance across individual samples. These clusters were not used for further analysis of protein expression. Cluster 1, 5 and 8 did show expression patterns correlated to the hierarchical grouping, indicating these protein expression patterns are clustered based on the differential expression in the different consumption phases and thus biologically relevant.

**Fig. 5:**
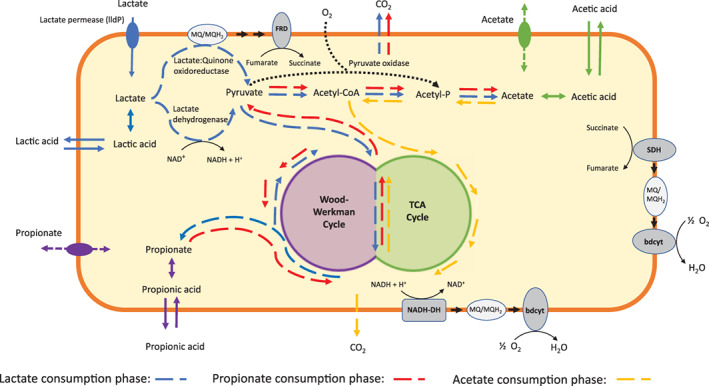
Schematic overview of the metabolism of lactate in *P*. *freudenreichii* in microaerobic conditions. Undissociated acids can be transported through passive diffusion (closed arrows across the membrane). Dissociated acids have to be transported by (putative) transporters (dashed arrows with closed circle). Lactate is metabolized in three phases: a lactate consumption phase, propionate consumption phase and acetate consumption phase. In the lactate consumption phase, lactate is metabolized to propionate and acetate. In the propionate consumption phase the ‘propionate switch’ occurs and the produced propionate is metabolized to acetate. In the acetate consumption phase the ‘acetate switch’ occurs and acetate is metabolized to CO_2_. Several options have been proposed for the production of Acetyl‐CoA from pyruvate. Either a pyruvate dehydrogenase complex (producing NADH) or pyruvate ferredoxin oxidoreductase (producing reduced ferredoxin) catalyses the conversion of pyruvate to Acetyl‐CoA. Recently, a ferredoxin‐based energy conservation system has been suggested in propionic acid bacteria (Marcellin *et al*., 2020) consisting of FixABCX, which oxidizes reduced ferredoxin and a quinol by reducing 2 NAD^+^ (Ledbetter *et al*., 2017; Marcellin *et al*., 2020). The entire complex (FixABCX) could also be reconstituted in our proteomics data, supporting the possible role of pyruvate‐ferredoxin oxidoreductase in the synthesis of acetyl‐CoA from pyruvate and maintaining redox balance for anaerobic growth on lactate. A pyruvate oxidase (black dotted line), producing acetyl‐phosphate from pyruvate and phosphate while consuming oxygen, has also been identified in the genome of *P*. *freudenreichii*, which may function as an energy‐yielding oxygen tolerance mechanism (Marcellin *et al*., 2020) and which may explain lower propionate:acetate ratios found in microaerobic conditions compared to anaerobic conditions in *P*. *freudenreichii*. Two gene clusters of the succinate dehydrogenase/fumarate reductase can be found in the genome of *P*. *freudenreichii*, *sdhABC1* (RM25_1246‐RM25_1248) and *sdhA3B3C2* (RM25_1350‐RM25_1352). Most likely, one copy of the succinate dehydrogenase genes acts as fumarate reductase whilst the other copy acts as succinate dehydrogenase (Brzuszkiewicz *et al*., 2011; Parizzi *et al*., 2012). FRD, fumarate reductase; SDH, succinate dehydrogenase; bdcyt, bd‐type cytochrome; NADH‐DH, NADH dehydrogenase; MQ, menaquinone; MQH_2_, menaquinol. [Color figure can be viewed at wileyonlinelibrary.com]

##### Anaerobic and respiratory pathways are continuously expressed in anaerobic and microaerobic conditions

In both anaerobic and microaerobic conditions expression of the complete pathways for metabolism of lactate to propionate and acetate were detected. We detectedl‐lactate permease (LldP), lactate dehydrogenase (NADH dependent) (Ldh1, Ldh2) and a complex of three proteins which has been linked to lactate oxidation coupled to fumarate reduction by quinones or to cytochromes (Lactate utilization protein (LldE, LldF, LldG)) (Pinchuk *et al*., 2009).

All proteins in the Wood–Werkman cycle were detected. Pyruvate‐flavodoxin oxidoreductase (Nifj1), pyruvate oxidase (RM25_0410) and pyruvate dehydrogenase (AceE, PdhB, BkdA1) were found, together with phosphate acetyltransferase (Pta) and acetate kinase (AckA) completing the pathway for acetate production. Interestingly, proteins involved in the Wood–Werkman cycle were as abundant in the propionate consumption phase compared to that in lactate consumption phase, and no upregulation of other enzymes known to be involved in bacterial propionate metabolism was found, supporting the hypothesis of the Wood–Werkman cycle running in reversed direction as the pathway for propionate oxidation, as previously suggested by Emde and Schink (1990).

The modified TCA cycle suggested by Beck and Schink (1995), menaquinone biosynthesis proteins, heme biosynthesis proteins and cytochrome bd oxidase subunit I (CydA) were all found to be expressed in anaerobic and microaerobic conditions. The expression of cytochrome bd oxidase remained stable in anaerobic and microaerobic conditions. Cytochrome bd is characterized by a high affinity for O_2_ and is preferentially expressed at low O_2_ tension (Tseng *et al*., 1996; Giuffrè *et al*., 2014) but is also expressed at similar levels in anaerobic conditions in several bacteria (Govantes *et al*., 2000; Machado *et al*., 2006). Cytochrome bd oxidase is clearly linked to conditions in which the high affinity to oxygen (D'mello *et al*., 1996; Baughn and Malamy, 2004) of cytochrome bd oxidase becomes beneficiary for the scavenging of oxygen. The expression of cytochrome bd oxidase in *P*. *freudenreichii* even in anaerobic conditions suggests that *P*. *freudenreichii* is prepared for the availability of O_2_. In line with our results, in *Cutibacterium acnes* the whole respiratory chain was also expressed in anaerobic conditions (Brzuszkiewicz *et al*., 2011). Proteins needed for respiration processes are thus abundant in anaerobic conditions in the absence of additional external electron acceptors. We hypothesize this behaviour reflects adaptation to an environment in which variable but low supply of oxygen prevails. In such an environment, possession of an electron transport chain with a high affinity for oxygen is beneficial, while such an enzyme system does not need to cope with high concentrations of molecular oxygen. This hypothesis fits the observations that oxygen is toxic at high concentrations (de Vries *et al*., 1972) but at low rates can improve biomass production (this study and Pritchard *et al*. (1977).

##### Microaerobic propionate and acetate consuming cells show distinct proteomes compared to lactate grown cells

The proteins involved in the conversion of lactate to pyruvate, Wood–Werkman cycle, acetate production and TCA cycle were not found to be significantly different in abundance when comparing anaerobic and microaerobic conditions for cells growing on lactate. Hierarchical clustering revealed proteomes were highly similar between anaerobic and microaerobic lactate‐grown cells (Fig. 4). Only 12 proteins were found to be differentially expressed, supporting the evidence for similar expression patterns amongst lactate‐grown cells. This points towards fermentative behaviour whenever lactate is present, even in microaerobic conditions, which is confirmed by the production of propionate.

Cells growing on propionate and acetate show distinct proteomes from lactate grown cells. This is reflected by the higher amount of significantly different expressed proteins compared to microaerobic lactate consuming cells (43 for propionate consuming cells and 70 for acetate consuming cells (Fig. 3)). Clear protein clusters with different expression patterns between lactate‐grown and propionate or acetate‐grown cells were revealed using k‐means clustering (cluster 1, 5 and 8).

##### Aerobic respiration is upregulated in propionate and acetate consuming cells

Cluster 1 consists of proteins upregulated in microaerobic propionate and acetate‐grown cells compared to anaerobic and microaerobic lactate‐grown cells. Notably, the cluster contains proteins involved in pyruvate metabolism (pyruvate dehydrogenase, pyruvate ferredoxin oxidoreductase), as well as ferredoxin itself, indicating higher activity towards production of acetate (Marcellin *et al*., 2020). Several proteins in the TCA cycle were also grouped in cluster 1: Citrate (si)‐synthase (GltA1, GltA2), citrate(isocitrate) hydro‐lyase (AcnA), succinate‐semialdehyde dehydrogenase (GabD). This indicates further upregulation of proteins involved in the TCA cycle in cells growing on propionate and acetate. Cytochrome bd oxidase subunit I and NAD(P)H:quinone oxidoreductase, which were found to be significantly differently expressed (Fig. 3), were also present in this cluster, in line with further activation of aerobic electron transport. Cytochrome P450 was also present in this cluster. Cytochrome p450 is a b‐type cytochrome (Munro and Lindsay, 1996) containing heme, involved in oxidative metabolism of a wide variety of substrates (Omura, 2005). NADH:flavin reductase was significantly more abundant in cells growing microaerobic on lactate compared to anaerobic conditions and was also significantly more abundant in acetate‐grown cells compared to microaerobic lactate‐grown cells. NADH:flavin reductase regenerates NAD^+^ by reduction of flavins with NADH (Fontecave *et al*., 1987). Energy production and reoxidation of NADH depend greatly on flavin‐dependent enzymes in aerobic conditions (Vorobjeva, 1999). These findings point towards further upregulation of aerobic respiration in microaerobic conditions in propionate and acetate consuming cells.

##### Microaerobic conditions trigger oxidative stress response

Cells growing in microaerobic conditions showed upregulation of oxidative stress‐related proteins. FrnE, a chaperone protein from the DSBA oxidoreductase family that protects proteins from oxidation during oxidative stress (Khairnar *et al*., 2013), was significantly upregulated in cells growing on lactate in microaerobic conditions compared to anaerobic conditions. NrdJ, a vitamin B_12_‐dependent ribonucleotide reductase involved in DNA repair in aerobic conditions was upregulated in the microaerobic lactate consumption phase compared to acetate and propionate consumption phase. Interestingly, in the acetate consumption phase several proteins which have been linked to bacterial oxidative stress response in various bacteria, glutathione S‐transferase (*P*. *freudenreichii*) (Falentin *et al*., 2010), FeS cluster assembly protein SufB (*E*. *coli*) (Outten *et al*., 2003) and two component response transcriptional regulatory protein MprA (*Mycobacterium tuberculosis*) (He *et al*., 2006) were less abundant compared to microaerobic lactate grown cells. This indicates *P*. *freudenreichii* cells growing microaerobically on lactate experience higher oxidative stress levels compared to cells consuming acetate. Oxidative stress from radical formation largely depends on the activity of electron transport chains and is highest in the exponential growth phase (González‐Flecha and Demple, 1995). The decreased abundance of oxidative stress response proteins may thus be a result of a decreased activity of electron transport chains due to the lower growth rates caused by the slow consumption rates of propionate and acetate. This is supported by cluster 8, consisting of proteins that were most abundant in anaerobic and microaerobic lactate‐grown cells, average abundant in propionate‐grown cells and least abundant in acetate‐grown cells. Several proteins involved in heme (HemA, HemY, HemK) biosynthesis were found in this cluster, indicating a decreased biosynthesis of heme, which is needed for aerobic respiration. Interestingly, also proteins involved in vitamin B_12_ synthesis (CobA, CbiF, CbiM, CbiN, CbiQ, CbiL, CbiX, CobU) were found in this cluster, pointing towards lower demands for vitamin B_12_ in the acetate consumption phase, in line with the decrease of vitamin B_12_‐dependent NrdJ.

##### Lower growth rates on propionate and acetate are reflected by the proteome

The large difference in growth rates on lactate (either anaerobic or microaerobic) and propionate or acetate was reflected by proteomic clustering, which revealed a cluster with proteins with higher abundance in lactate‐grown cells, average abundance in propionate‐grown cell and lower abundance in acetate‐grown cells (cluster 5). The main proteins in this cluster were related to biosynthesis processes: the 30S and 50S ribosomal proteins, DNA/RNA polymerase, aminoacyl‐tRNA ligases (protein synthesis) and elongation factors. This reflects the higher growth rates in cells growing on lactate, either anaerobic or microaerobic, compared to propionate and acetate. This was supported by significant downregulation of several proteins related to transcription (HrdD, RmlN (Hansen *et al*., 2008)), synthesis of purines, thymidylate and methionine (5‐formyl tetrahydrofolate cyclo‐ligase (Hansen *et al*., 2008)), sulfur amino acid biosynthesis (sulfate adenylyltransferase (Ullrich *et al*., 2001)) and active growth (methionine aminopeptidase (Shapiro *et al*., 2011) and RtcB (RNA ligase (Chakravarty *et al*., 2012))) in acetate consuming cells. Together, these results indicate that *P*. *freudenreichii* cells consuming acetate impair their DNA, RNA and nucleotide synthesis and activate systems to become dormant and persistent, a survival strategy activated by a range of microorganisms including *E*. *coli* (Lewis, 2005).

### Biological niche propionic acid bacteria

Natural habitats of bacteria often include transition zones between oxic and anoxic environments. When diffusion of oxygen is lower compared to the consumption of oxygen by the microbial community, a transition zone with O_2_ concentrations between oxic and anoxic levels exist (Morris and Schmidt, 2013). Such environments with microoxic zones include soil aggregates (Tiedje *et al*., 1984), sediments, the gastrointestinal tract of animals (Morris and Schmidt, 2013) and the human gut (Albenberg *et al*., 2014). Bacteria thriving in these environments often contain cytochrome oxidases of the bd type, which have high affinity for oxygen (D'mello *et al*., 1996; Baughn and Malamy, 2004) and therefore are functional in microoxic environments (Morris and Schmidt, 2013) by enabling aerobic metabolic flux at extremely low oxygen pressures (Puustinen *et al*., 1991). PAB contain bd‐type cytochromes and are commonly found in soil (Hayashi and Furusaka, 1979), silage (Merry and Davies, 1999), the rumen of animals (Bryant, 1959) and human intestines (Albenberg *et al*., 2014), which contain microoxic zones. In the gut diffusion of oxygen from epithelial cells creates a low constant flux of oxygen which is consumed by the gut microbiota in the mucosa (Morris and Schmidt, 2013). Modulation of the pO_2_ of the gut increased the abundance of *Actinobacteria*, including *Propionibacterium*, showing that these microorganisms benefit from low concentrations of oxygen being present (Albenberg *et al*., 2014). In our chemostat reactor the optimum gas inflow concentration contained a pO_2_ of 16–32 mm Hg, close to the baseline levels of pO_2_ of 40 mm Hg found in human intestinal tissues samples (Albenberg *et al*., 2014). Our results showed that at low concentrations of oxygen indeed PAB grow and benefit from oxygen. When oxygen is available, *P*. *freudenreichii* can utilize fermentation products using the electron transport chain. In this way, *P*. *freudenreichii* ensures maximum uptake of substrates. The enzymatic activities needed in anaerobic and microaerobic metabolism are largely overlapping, making it possible to consume propionate and acetate with limited further investments in terms of *de novo* protein synthesis. The ability to scavenge fermentation end products of other microorganisms in microbial communities, like lactate, propionate, acetate and propanediol (Saraoui *et al*., 2013) further links to microoxic zones, like the gut, rumen and soil as ecological niche for PAB. At lower pH regions in the gastrointestinal tract, like the caecum with a pH of 5.7 (Fallingborg, 1999), passive diffusion of undissociated acids into the cell may occur. However, in more neutral regions of the gastrointestinal tract (pH up to 7.4 in the terminal ileum (Fallingborg, 1999)) or in the rumen (pH up to 6.5 (Argyle and Baldwin, 1988)) *P*. *freudenreichii* would greatly benefit from the presence of active propionate and/or acetate transporters. The ability to actively import these compounds using specific transporters instead of relying on passive diffusion needs to be investigated for *P*. *freudenreichii*.

## Conclusion

Here we have shown PAB can greatly benefit energetically from aerobic respiration via electron transport chains with oxygen as terminal acceptor. We have shown that under low levels of oxygen supply, *P*. *freudenreichii* deploys fermentative behaviour on lactate via the Wood–Werkman cycle and is able to subsequently oxidize its fermentation products propionate and acetate in a preferred order, pointing to an extension of its metabolic repertoire. The switch from the production of propionate to consumption of propionate is a phenomenon we propose to coin as the ‘propionate switch’ in analogy to ‘acetate switch’ described in *E*. *coli* (Wolfe, 2005).

The ability of *P*. *freudenreichii* to utilize fermentation end products secreted by other microbes, next to lactate, now also propionate and acetate, in combination with a functional respiratory chain containing a high O_2_ affinity cytochrome bd complex, points towards a niche occupation in microoxic environments like the rumen and human intestine. The expression of all redox proteins, electron carriers and electron transport chain proteins in anaerobic conditions further support the hypothesis of the ecological niche of *P*. *freudenreichii*. The impact of (an)aerobic electron transfer chains on PAB ecology and colonization of various ecosystems with low supply of oxygen requires further studies.

## Experimental procedures

### Strains and media

Sixteen strains of *Propionibacteriaceae* spp. (six strains *Propionibacterium freudenreichii*, two strains *Acidipropionibacterium thoenii*, five strains *A*. *jensenii* and three strains *A*. *acidipropionici*) isolated from different dairy sources were obtained from the public culture collections DSMZ or BCCM (see Supplementary File 1). All strains were initially cultivated using YEL containing per litre: 12.8 g l‐lactic acid (16 g 80% l‐lactic acid syrup, Sigma‐Aldrich), 10 g tryptone (Oxoid), 5 g yeast extract (Oxoid), 5 g potassium dihydrogen phosphate. pH was adjusted to 7.0 using 5 M NaOH prior to autoclaving at 121°C for 15 min. Strains were stored in 30% (vol./vol.) glycerol cryovials at −80°C.

### Culture conditions initial screening

Strains were streaked from −80°C stocks on YEL plates and incubated for 7 days at strict anaerobic conditions using anaerobic gas (gas mixture: 80% N_2_, 10% CO_2_, 10% H_2_) and an oxygen catalyst at 30°C in anaerobic jars. For aerobic incubation, single colonies were inoculated in 10 mL YEL in 100 mL shake flasks (±1 cm culture layer thickness) and incubated at 30°C static and shaking (120 rpm) for 5 days. For anaerobic incubation, single colonies were inoculated in 10 mL YEL in 50 mL Greiner tubes. Anaerobic samples were incubated at 30°C for 5 days in anaerobic jars at strict anaerobic conditions using anaerobic gas (gas mixture: 80% N_2_, 10% CO_2_, 10% H_2_) and an oxygen catalyst at 30°C in anaerobic jars. The optical density was measured at 600 nm (OD_600nm_).

### Long‐term batch cultivation

A single colony of *P*. *freudenreichii* DSM 20271 was inoculated in 10 mL YEL and incubated at 30°C anaerobically for 2 days, after which 1% (vol./vol.) was inoculated into bioreactors with a working volume of 500 mL (Multifors, Infors HT, Switzerland). The stirring speed was set at 300 rpm, the temperature was kept constant at 30°C and the pH was controlled at 7.0 by automatic addition of 5 M NaOH and 0.5 M HCl. The gas mix containing N_2_ gas and air was supplied through a sparger at the bottom of the fermenter using a mass flow controller premixing gas at set values at a rate of 0.1^−1^min L*. Cultivations were followed for a period of 400 h. 10 mL samples were taken at different intervals.

### Biomass quantification

Biomass was quantified by measuring the cell dry weight (CDW) concentration. Briefly, weighed samples were passed through pre‐weighted membrane filters with a pore size of 0.2 μm (Pall Corporation, Ann Arbor, MI, USA) by a vacuum filtration unit. Residual cell material was washed using demi water. Filters were dried at 80°C for 2 days and weighed to determine the CDW concentration in g* kg^−1^culture. For yield calculations CDW values were calculated from OD_600nm_ values using a second‐order polynomial relation (van Mastrigt *et al*., 2018a).

### Analysis of extracellular metabolites

Lactate, acetate and propionate were quantified by high‐performance liquid chromatography as described by van Mastrigt *et al*. (2018b).

### Proteome analysis

#### Proteomic sample preparation and analysis

*P. freudenreichii* cells grown in the long‐term batch cultivation were collected in 1 mL tubes and the cell pellet was frozen at −80°C. Samples were taken at 30 h for anaerobic conditions. In microaerobic conditions, samples were taken after 30 h (lactate consumption phase), 5 or 7 days (propionate consumption phase) and between 13 and 15 days (acetate consumption phase). Samples were washed twice with 100 mM Tris–HCl (pH 8) and resuspended in 100 μl 100 mM Tris–HCl. Samples were lysed by sonication for 45 s twice while cooling 1 min on ice in‐between. The protein content was determined using Pierce Coomassie protein assay and samples were diluted to 1 μg*μl^−1^ using Tris–HCl buffer (pH 8). Samples were prepared according to the filter assisted sample preparation protocol (Wiśniewski *et al*., 2009) with the following steps: reduction with 15 mM dithiothreitol, alkylation with 20 mM acrylamide and digestion with sequencing grade trypsin overnight. Each prepared peptide sample was analysed by injecting (5 μl) into a nanoLC‐MS/MS (Thermo nLC1000 connected to a Q Exactive HFX Orbitrap) as described previously (Lu *et al*., 2011). nLC‐MSMS system quality was checked with PTXQC (Bielow *et al*., 2016) using the MaxQuant result files. LCMS data with all MS/MS spectra were analysed with the MaxQuant quantitative proteomics software package (Cox *et al*., 2014) as described before (Smaczniak *et al*., 2012; Wendrich *et al*., 2017).

#### Proteome data filtering, statistics and analysis

A protein database with the protein sequence of *P*. *freudenreichii* DSM 20271 (ID:UP000032238) was downloaded from UniProt. Filtering and further bioinformatics and statistical analysis of the MaxQuant ProteinGroups file were performed with Perseus (Tyanova *et al*., 2016). Reverse hits and contaminants were filtered out. Protein groups were filtered to contain minimally two peptides for protein identification of which at least one is unique and at least one is unmodified. Also, each group required three valid values in at least one of the experimental groups. Volcano plots were prepared based on the Student's *t*‐test difference between samples. Volcano plots were produced in Rstudio (Racine, 2012) using EnhancedVolcano (Blighe *et al*., 2018). Proteins were considered to be significantly different amongst sample groups if *P* < 0.01 and at least a fourfold change difference was detected. A heatmap was constructed using Pheatmap (Kolde, 2015) in R‐studio. LFQ intensity values were normalized using *Z*‐scores (Jain *et al*., 2005), after which hierarchical clustering was performed amongst samples using complete‐linkage clustering in Pheatmap. Protein expression pattern analysis and consequent clustering based on expression pattern was performed by K‐means partitioning (MacQueen, 1967) in Pheatmap using *k* = 8, determined by the gap statistic method (Tibshirani *et al*., 2001) and the elbow method using factoextra (Kassambara and Mundt, 2017) in Rstudio.

## Supporting information

**Appendix S1:** Supplementary Information
