# Supplementary material for: Propionibacterium freudenreichii thrives in microaerobic conditions by complete oxidation of lactate to CO2
Source: Environ Microbiol. 2021 May 6;23(6):3116–29. doi: 10.1111/1462-2920.15532 (PMC8360058; doi:10.1111/1462-2920.15532)
Supplement: Supplementary file 1 — Appendix S1: Supplementary Information [file EMI-23-3116-s001.zip › EMI_15532_Supplementary_figure_1.pdf]

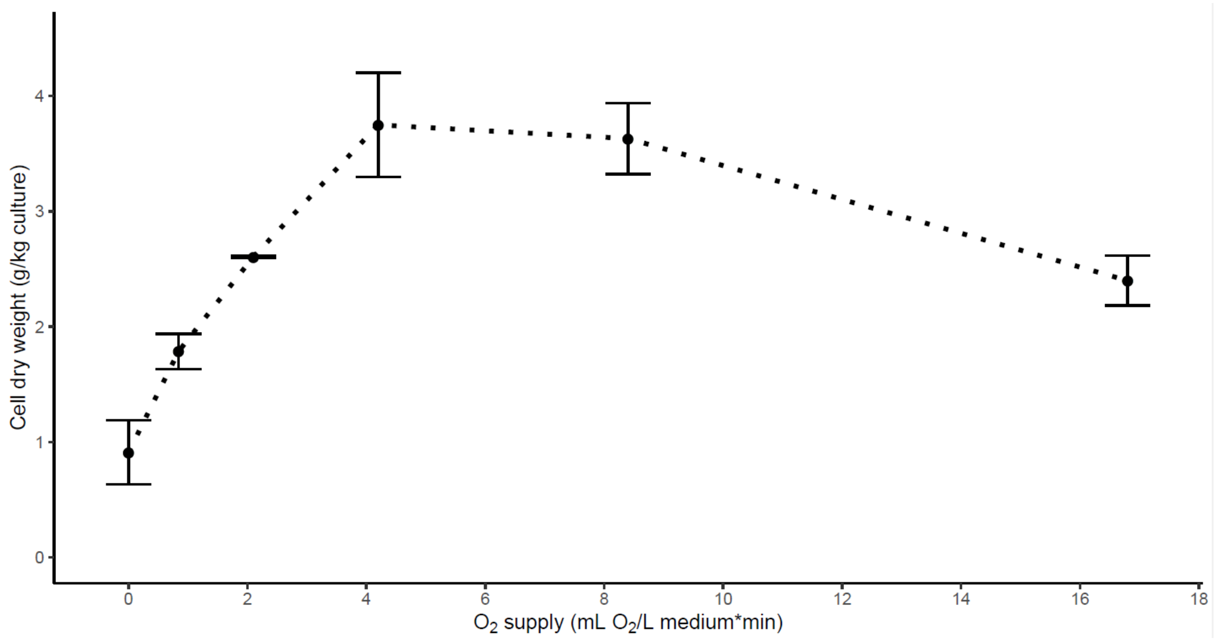

Supplementary figure 1: Biomass formation at various O_2 supply rates. Chemostat cultivations were performed in bioreactors with a working volume of 500 mL. A constant dilution rate of 0.1 was used. pH was kept constant at 7.0 by automatic addition of HCl and NaOH. A stirring speed of 300 rpm and temperature of 30°C degrees was used. Gas was supplied at the bottom of the reactor using a gas sparger at a constant rate of 0.1 L/min. Nitrogen and air were mixed in defined ratios in a mass flow controller prior to entering the bioreactor through 0.2 µm sterile filters.
