# Supplementary material for: Propionibacterium freudenreichii thrives in microaerobic conditions by complete oxidation of lactate to CO2
Source: Environ Microbiol. 2021 May 6;23(6):3116–29. doi: 10.1111/1462-2920.15532 (PMC8360058; doi:10.1111/1462-2920.15532)
Supplement: Supplementary file 1 — Appendix S1: Supplementary Information [file EMI-23-3116-s001.zip › EMI_15532_Supplementary_figure_2.pdf]

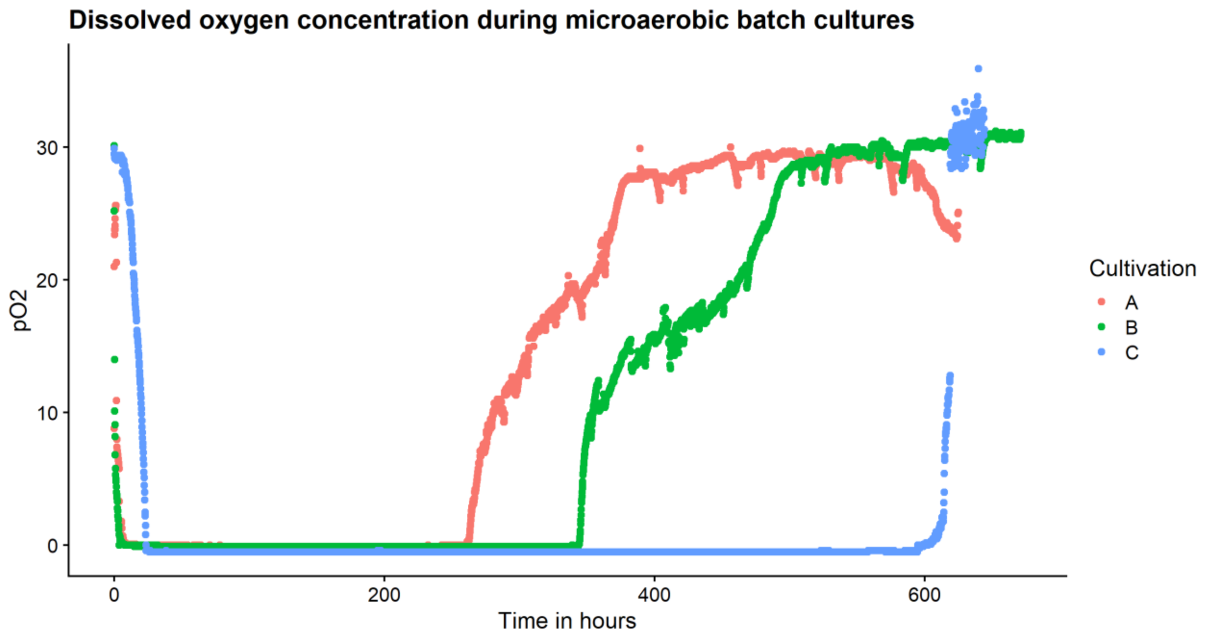

Supplementary figure 2: Dissolved Oxygen levels measured during prolonged batch cultures of P. freudenreichii subsp. freudenreichii DSM 20271 in bioreactors. Time = 0 represents the time at which the reactors were inoculated. Sensors were calibrated to 0% and 100% by using two points: 100% was achieved by supplying pure air using the same stirrer speed, temperature, flow rate and working volume as used during fermentation, 0% was achieved by supplying pure nitrogen gas using the same settings
